# Practice guidelines for the supervising professional: intraoperative neurophysiological monitoring

**DOI:** 10.1007/s10877-013-9496-8

**Published:** 2013-09-11

**Authors:** Stanley A. Skinner, Bernard Allan Cohen, David Eric Morledge, John J. McAuliffe, John Daniel Hastings, Charles D. Yingling, Michael McCaffrey

**Affiliations:** 1 Neurological Monitoring Associates, LLC, 333 West Brown Deer Road, MS #240, Milwaukee, WI 53217-4925 USA; 2 Neurophysiology Department, Abbott Northwestern Hospital, Piper Building, 800 E 28th Street, Mail Route 39304, Minneapolis, MN 55407 USA; 3 Neurostatus, 5120 W Overland Rd., PMB 239, Boise, ID 83705 USA; 4 Neurologic Medicine PLLC, 5563 S. Lewis Avenue, Suite 100, Tulsa, OK 75105 USA; 5 Department of Anesthesia, Cincinnati Children’s Hospital Medical Center, The University of Cincinnati, 3333 Burnet Ave., Cincinnati, OH 45229 USA; 6 Clinical Professor, Otolaryngology, Stanford School of Medicine, 1001 Bridgeway #434, Sausalito, CA 94965 USA; 7 Biotronic NeuroNetwork, 812 Avis Drive, Ann Arbor, MI 48108 USA

**Keywords:** The American Society of Neurophysiological Monitoring, Intraoperative neurophysiological monitoring, Supervision, Guidelines

## Abstract

The American Society of Neurophysiological Monitoring (ASNM) was founded in 1988 as the American Society of Evoked Potential Monitoring. From the beginning, the Society has been made up of physicians, doctoral degree holders, technologists, and all those interested in furthering the profession. The Society changed its name to the ASNM and held its first Annual Meeting in 1990. It remains the largest worldwide organization dedicated solely to the scientifically based advancement of intraoperative neurophysiology. The primary goal of the ASNM is to assure the quality of patient care during monitored procedures along the neuraxis. This goal is accomplished through programs in education, advocacy of basic and clinical research, and publication of guidelines. The ASNM is committed to the development of medically sound and clinically relevant guidelines for intraoperative neurophysiology. Guidelines are formulated based on exhaustive literature review, recruitment of expert opinion, and broad consensus among ASNM membership. Input is likewise sought from sister societies and related constituencies. Adherence to a literature-based, formalized process characterizes the construction of all ASNM guidelines. The guidelines covering the Professional Practice of intraoperative monitoring were established by a committee of nearly 30 total participants and ultimately endorsed by the Board of Directors of ASNM on January 24th 2013. That document follows.

## Introduction

These Guidelines directly apply to supervising practitioners who render professional Intraoperative Neurophysiological Monitoring (IONM) services in the United States of America. An additional objective of these Guidelines is to provide the American public with information about the roles and responsibilities of professionals in the execution of their supervisory duties.

IONM is the use of physiological techniques (1) to assess neural integrity and/or (2) to map or neuro-navigate within at-risk neural structures during surgical procedures. The field of IONM is currently practiced under a number of different models [6, 11–14]. While recognizing this complex framework, a set of core unified Guidelines can promote best practice patterns. The Guidelines are based upon the tenet that the Professional’s delivery of IONM services constitutes a patient care activity and thus establishes a relationship between the provider and the patient [7]. The Guidelines apply without regard to the physical location of the Professional.

These Guidelines attempt to unify present models into an approach that:

- respects patient autonomy,
- optimizes intraoperative situational awareness, and
- reinforces collegiality among co-practitioners.

The authors recognize that time and effort will be required for many providers to achieve the level of IONM Professional care detailed within this document. The Guidelines are being released during an era of uncertain health care economics; therefore, no time horizon for full implementation is prescribed or suggested. However, basic patient-physician/practitioner interactions, such as identification of the IONM Professional to the patient, should be instituted immediately [4, 9].

Given rapidly evolving technologies and improvements in healthcare delivery systems, these Guidelines will be reviewed periodically to incorporate the latest information and science available [10].

## Abbreviations and definitions

IONM: Intraoperative Neurophysiological Monitoring
IONM-P: Intraoperative Neurophysiological Monitoring Supervising Professional
IONM-T: Intraoperative Neurophysiological Monitoring Technologist
QHP: Qualified Healthcare Professional
Physician: A physician with a State license to practice medicine at the clinical site
Proceduralist: A surgeon or other interventionalist

## Necessity for IONM-P practice Guidelines

The practice of IONM is a patient care activity; therefore, the Guidelines embrace the principle that IONM must be patient centered. A traditional patient-physician/practitioner relationship exists whether or not the IONM-P is a physician or other qualified healthcare professional practitioner. This document:

- defines the scope of that relationship,
- recommends ways to protect the interests of the patient,
- informs, instructs, and enlightens IONM-Ps at all levels of experience,
- provides a coherent explanation of the IONM discipline to the larger universe of healthcare providers.

These Guidelines are intended to benefit hospital credentialing committees, licensing bodies, regulators, payors, health care societies, surgeon or proceduralist societies, and others who request guidance on IONM standards of practice.

As a patient care activity, IONM practice differs significantly from neurophysiological laboratory studies (EEG, EP, EMG) or instances of IONM restricted to brief epochs (e.g., surgeon or proceduralist request for pedicle screw stimulation only). In the case of laboratory testing or restricted IONM, the IONM-P is asked to identify possible neuropathophysiology at one moment in time. In contrast, during the course of complex multimodality IONM, the IONM-P is engaged in an activity that requires the IONM-P to be continuously available to intervene or supervise IONM patient care over a time period of many hours. Moreover, the patient–centered IONM philosophy enjoins IONM-Ps to identify themselves to patients and to explain the IONM-P’s specific role in advance of monitored procedures.

## IONM practice Guidelines not a medicolegal document

These Guidelines are an attempt to define minimum IONM-P practices under typical circumstances. Because each case has unique circumstances, the failure to completely meet some aspects of these Guidelines cannot be construed to imply negligence or breach of duty.

## IONM-P definition

The Intraoperative Neurophysiological Monitoring Professional (IONM-P) is the provider of real time technological supervision, interpretation, and diagnostic/therapeutic (interventional) suggestions or recommendations during IONM. There are three distinct components to IONM: *technological, interpretive, and diagnostic/therapeutic (interventional)*.

The *technological* component involves placement of appropriate electrodes, acquisition of high quality data, data recording, troubleshooting problems, and providing a description of the recordings. Knowledge of neuroanatomy, neurophysiology, neuropharmacology, the pre-operative status of the patient, and the scientific literature permits *interpretation* of recorded waveform data within the context of the surgical procedure. During mapping/neuronavigation or in the event of threatened neural integrity, surgical/anesthetic *intervention* is planned collaboratively with active participation (sometimes specific guidance) by the IONM-P. All three of these patient care components (technological, interpretive, and interventional) fall within the duties and responsibilities of the IONM-P However, the technological component may be carried out in whole or in part by an appropriately experienced/credentialed Intraoperative Neurophysiological Monitoring Technologist (IONM-T) [12].

IONM-Ps seeking guidance on the definition of “Professional” may refer to the *Corrections Document*—*CPT* ^®^ *2012* (American Medical Association Web Site [1] which states:

A “Physician or other Qualified Healthcare Professional” [(QHP)] is an individual who is qualified by education, training, licensure/regulation (when applicable), and facility privileging (when applicable) who performs a professional service within his/her scope of practice and independently reports that professional service. These professionals are distinct from “clinical staff”. A clinical staff member is a person who works under the supervision of a physician or other [QHP] and who is allowed by law, regulation and facility policy to perform or assist in the performance of a specified professional service, but who does not individually report that professional service. Other policies may also affect who may report specific services.”

Some components of professional supervision, as defined in these Guidelines, may be regulated according to States’ laws requiring specific certification or licensure. Each IONM-P must comply with local statutory authority concerning scope of practice and State licensure during delivery of patient care. Hospital credentialing of the IONM-P should include evidence of an appropriate combination of education, board certification, training, experience, and continuing education within the field of IONM (see “Appendix”).

In this regard, it must be clearly stated that the major purpose of these Guidelines is to assure patients, the surgeon or proceduralist, and the anesthesiologist that the IONM-P will use his/her full breadth of neurophysiological knowledge to accurately interpret changes in waveform data, immediately report any threat to neural integrity, differentially diagnose within the procedural context, and suggest specific interventions to redress evolving neural injury or threat of injury. The IONM-P’s scope of practice, state licensing, and/or hospital credentialing must permit the full duties of care as described: interpretation including identification of possible cause(s) and intervention.

## IONM-P responsibilities: pre-operative

IONM patient care includes preoperative patient evaluation, IONM planning, intraoperative care, postoperative patient follow-up, and the management of personnel and instrumentation that support these activities. Overall responsibility for IONM performance rests with the IONM-P. Almost all IONM care is either provided personally by an IONM-P or is provided by an IONM-T supervised by an IONM-P. The IONM-P may delegate appropriate technological monitoring tasks to the IONM-T(s). Such delegation should be specifically defined by the IONM-P and should also be consistent with State law or regulations and hospital medical staff policy. The following recommendations should be integrated and amplified within a Policy and Procedure Manual.

In order to achieve optimum patient care and safety, the IONM-P is responsible for the following:

### Management of personnel and instruments

The IONM-P often directs application of monitoring techniques performed by the supervised IONM-T. The IONM-P should assure the assignment of appropriately skilled personnel for each patient and procedure. Electrical safety and HIPAA compliance of all instrumentation shall be tested and maintained by the appropriate technical, “biomedical” service. The IONM-P, through inquiry and follow-up, confirms the proper functioning of all hardware, software programming, wired and wireless communication gear, special stimulators, and recording leads to be used in IONM procedures. When necessary, the IONM-P should report any malfunctions or failures of hospital communication networks to the appropriate hospital personnel.

### Pre-IONM evaluation of the patient

Patients have a right to know the identities and functions of their health care team members [5]. There exist multiple means for patients to form a patient/provider relationship with their IONM-P. These may include, but are not limited to, introductions by personal/virtual interaction, telephone communication, letter/electronic mail, or referral to the IONM-P’s interactive website. The selected means of introduction should permit the patient to (1) identify the IONM-P(s) who will be (or may become) involved in their care and (2) to discuss, if desired, the IONM-P’s qualifications to provide that care. In addition, the patient should be able to easily obtain answers to questions about the techniques to be employed in addition to possible risks and benefits of the monitoring, etc. The means and timing of this pre-operative introduction are determined by the IONM-P’s preferred style of practice and the constraints of the institution in which the procedure takes place. Nevertheless, the duty of care (establishment of a patient-physician/practitioner relationship) is respected no matter the location of the IONM-P.

A Pre-IONM patient evaluation permits the development of an IONM care plan that considers patient factors that may influence the results, interpretation, and reliability of IONM [7]. Patient evaluation may include, for example, review of the medical record, history taking, focused examination (directly or virtually via telemedicine), description of IONM procedures (and their risks), rehearsal of wake-up test if pertinent, and overall assurance of informed decision making on the part of the patient. Although the IONM-T may contribute to the preoperative collection and documentation of patient data, the IONM-P is responsible for the individualized evaluation.

The Guideline authors recognize that there will be unusual circumstances in which it is not possible for the IONM-P to interact directly with the patient (see Sect. 7.3). Under these situations, the IONM-P may delegate some of these duties to an appropriately qualified individual.

### Prescribing the IONM plan

The IONM-P is responsible for formulating an individualized IONM patient care plan aimed at the greatest safety and highest quality for each patient. Before each case, the IONM-P confirms that the IONM-T understands the IONM strategy: the neurophysiological monitoring modalities to be performed, what types of monitoring changes may be expected, the alarm criteria for each modality, and any special stimulators/modalities for mapping/neuronavigation. Whenever possible, and in particular when great case complexity dictates, the IONM-P:

1. Reviews pre-operative imaging and/or imaging results.
2. Discusses with the surgeon or proceduralist specific operative risks and pre-plans tactics in the event of IONM signal change or pre-plans means to assess neural topography.
3. Discusses with the anesthesiologist the plan for induction and maintenance of anesthesia, management of neuromuscular blockade at various points, such as intubation, exposure, or relaxant avoidance, and the possibility of a wake-up test.

## IONM-P responsibilities: conduct of IONM

### Critical duties

The IONM-P:

1. Ensures that data essential for evaluation of the patient is available and that all equipment to be used for IONM is in proper condition;
2. Advocates for anesthetic conditions that optimize the likelihood of obtaining high quality IONM data within the constraints of the patient’s physiology;
3. Evaluates and interprets all baseline signals and requests changes in the monitoring procedures, if required;
4. Interprets all significant changes from baseline recordings (or responses obtained from topographical studies) in real time;
5. In real time, evaluates context appropriate data and recommends (or suggests) therapeutic interventions as indicated.

### Communications

*In order to assure proper Conduct of IONM, the IONM*-*P must be continuously available to interact with the:*

#### Surgeon or Proceduralist

The IONM-P:

1. Interprets IONM data changes within the procedural context and determines, to the extent possible, if alterations are related to surgeon or proceduralist activity, anesthetic effects, systemic patient variables, patient positioning, technical factors, or a combination of these:
  - Adverse alterations in neuromonitoring data that are interpreted to be possibly procedure-related should be reported as soon as possible, once reasonable suspicion of an impending neurological insult exists.
  - Data change of ambiguous origin (often artifactual) will be reported to the surgeon or proceduralist as needed and appropriate. This report will parallel attempts to further elucidate and correct the causative factor(s). Retrospective, or “after-the-fact” interpretation and reporting, is of negligible benefit to the patient or the surgeon.
2. Helps to determine and execute a plan of intervention to recover neural function when an adverse alteration in neuromonitoring data presents.
3. Selects the correct assessment techniques to answer anatomic, functional, or prognostic questions related to neural structures. These neural structures include eloquent cerebral cortex, subcortical nuclear sites during DBS implantation, cranial nerves/nuclei during posterior fossa surgery, spinal sensory/motor/reflex systems, and elements of plexi, cauda equina, peripheral nerve, etc.

#### Anesthesiologist

The IONM-P:

1. Conveys to the Anesthesiologist what IONM modalities are planned and what anesthetic strategies are recommended for successful monitoring during the procedure.
2. Advocates for maintenance of an anesthetized state appropriate to the successful monitoring of planned IONM modalities.
3. Communicates concerns related to neuromonitoring data that suggest neurologic compromise that could be related to patient positioning factors.
4. May suggest alterations to anesthetic regimens and/or physiologic measures when these appear to compromise neural integrity and/or IONM effectiveness. Examples include increasing cardiac output with appropriate pressors when MEPs are lost during hypotensive episodes, lowering inhalational agents in favor of intravenous agents when IONM signals are of inadequate signal/noise ratio for effective monitoring, or increasing concentrations of anesthetic agents when non-specific increases in EMG activity suggest premature awakening or imminent patient movement.
5. May request additional information related to devices and physiologic measures such as perfusion pressure, core temperature, blood gas and electrolyte concentrations, etc. to aid in the interpretation of IONM data.
6. Recognizes that the Anesthesiologist must manage a complex array of physiological parameters. Thus, the extent to which the anesthetic requirements of IONM can be met must be balanced with the obligation of the Anesthesiologist to optimize overall patient safety and welfare.

#### The IONM-T

The IONM-P:

1. Directs the data acquisition, recording and stimulus parameter optimization, troubleshooting, and provides the interpretation of baseline data.
2. Directs necessary communication related to monitoring status.
3. Directs neural topography/neuro-navigation methods and provides interpretation, as appropriate.
4. Provides intraoperative education and mentoring regarding basic and applied intraoperative neurophysiology.

### IONM during concurrent cases; sole dedication of the IONM-P to IONM

In some practice models, it is anticipated that the IONM-P may monitor more than one case at a time. Nevertheless, IONM demands *concurrent* evaluation and management of supervised cases. It is difficult to globally define a safe maximum number of simultaneously monitored cases. Therefore, the IONM-P must judge the maximum capacity based on the mix of case complexity. It is understood that attention will be unevenly divided among cases of varying complexity and acuity. However, sufficient attention must be apportioned to each case such that all duties of the IONM-P are maintained for all cases. When evolving case acuity no longer permits adequate contemporaneous evaluation, the IONM-P should be prepared to properly carry out a transfer or “hand off” of professional responsibilities for one or more cases. In addition, external, poorly controllable factors can affect particular case management. These factors may include, but are not limited to, late add-ons, emergencies, cases “set up under the drapes,” particular surgeon or proceduralist directives, or variable data transfer or application bandwidth requirements. Only the IONM-P can determine if such outlier issues permit IONM participation in a particular case. Therefore, the IONM-P must make the determination of the case load based on an assessment of neural structure(s) at risk, the number/sophistication of requisite modalities (case complexity), the experience of the IONM-T s involved in the cases, and the occurrence of exceptional outlier case issues.

IONM demands constant attention, and the IONM-P should be solely dedicated to IONM. Therefore, the IONM-P does not engage in other clinical or other distracting activities during IONM supervision. If IONM case responsibility must be transferred, a safe hand-off protocol should be followed whenever possible. Examples of similar transfers of responsibility can be found in air-traffic control, anesthesia practices, and other clinical environments. Pertinent information, such as pre-operative evaluation, baseline studies, and intra-operative findings/complications, must be thoroughly communicated before completion of the responsibility transfer. Internet connectivity, or other IONM service operations, may be interrupted. A policy and procedure protocol should be developed that includes appropriate notification of the surgeon or proceduralist when connectivity is disrupted.

It is further recognized that cases of greater complexity may require continuous personal attendance within the operating room. This determination should be made well in advance of the day of a non-emergent surgery. After appropriate preoperative assessment and consultation with the surgeon or proceduralist as needed, the IONM-P will determine the best type of attendance to minimally meet the three basic responsibilities (supervision, interpretation, and discussion of intervention options). Consequently, the minimal attendance requirement may be met by telecommunications alone, by telecommunications with standby personal attendance, or by planned personal attendance throughout the procedure.

### IONM by surgeons or proceduralists

In limited circumstances, surgeons/proceduralists may record and interpret IONM data in areas where they hold expert understanding of the associated neurophysiological principles. Such cases may include facial or recurrent laryngeal nerve monitoring by otolaryngologists, pedicle screw assessment by spine surgeons or proceduralists, or basic neural topography by anesthesiologists doing nerve blocks. All such instances assume documented training by the surgeon or proceduralist in the circumscribed applied technique. Each hospital should develop program policies, procedures, and credentialing to determine the appropriateness of surgeon or proceduralist IONM data recording and/or supervision.

### Post-IONM evaluation of the patient

The IONM-P’s duty to the patient may not end with completion of surgery. Chart review, discussion with the surgeon or proceduralist, or personal follow-up may be necessary to answer postoperative questions. False negative and false positive reporting can occur during IONM. Post-IONM quality assurance methods should be part of an IONM program to analyze IONM-P and IONM-T performance, appropriateness of IONM procedures, and function of IONM instrumentation, etc. The sum of this data permits the generation of appropriate quality assurance (QA) and quality improvement (QI) reports, and the formulation of outcomes measures.

## Documentation and reports

It is important to appropriately document and archive recorded IONM data during all monitored surgeries as outlined in 8.1.3.

### IONM data recording

Representative samples from the sequence of recorded IONM modalities should be archived in order that the intraoperative course of that patient can be adequately reconstructed. All stimulus-evoked responses (SSEP, ABR, TcMEP, etc.) should be archived with an associated commentary. Because most instruments of recent vintage permit A/D conversion of EEG bandwidth, continuous EEG (during carotid endarterectomy, for example) should be archived. Otherwise, very frequent “screen saves” should be archived so that the case may be suitably reconstructed. Storage capacity limits for EMG currently prohibit a continuous data archive on many instruments. “Screen saves” of representative pathologic discharges (alerts) and recurring artifacts should be archived so that the case may be thoroughly understood by any reviewer at a later time. In the absence of such alerts, representative samples of ongoing EMG activity should be saved periodically.

### Physiological parameters

Blood pressure, core temperature, blood gas concentration, and rate of administration of relevant intravenous anesthetics should be observed, logged, and made available to the IONM-P at frequent intervals and at the occurrence of significant procedural/physiological events, such as an alert of monitored data change. Electrolyte concentrations, hematocrit, measures of intravascular volume, and many other physiological parameters are often helpful and should be reported and logged as appropriate. An electronic or printed log sheet should be maintained.

### Report

The report of each IONM session should include details of patient history, the surgical procedure(s) performed, the types of modalities recorded, description of the baseline responses, neural topographical/neuronavigational data acquired, details of any significant changes in responses during the procedure, interventional measures, closing responses obtained, and details of immediate post-operative findings where relevant. These reports are supplemented by description of any significant communications throughout the procedure between the IONM-P or IONM-T and the surgical and/or anesthesia team. All communications and reports are acquired, transferred, and archived under HIPAA compliant procedures.

## Education and quality assurance

Every IONM program needs to mature and improve over time, especially in response to changes in the field. In order for this to occur, it is important for each IONM program to provide for ongoing education and quality assurance. The IONM-P oversees these activities. QA/QI programs for IONM should be established. These programs must be supported by a robust data collection and analysis infrastructure so that patient outcomes can be monitored and improved over time.

## Ethical practice of IONM

The patient-IONM-P relationship involves special obligations for the IONM-P that includes placing the patient’s interests first and foremost, faithfully caring for the patient, and being truthful [4, 8, 15, 16]. To achieve these obligations the IONM-P will:

- respect the right of every patient to self-determination;
- recognize the right of the patient to know who the IONM-P is and what IONM contributes to the planned surgical procedure;
- understand the autonomy of the patient and recognize the responsibilities and obligations of a patient care activity;
- acknowledge and respect the vulnerability of anesthetized patients;
- strive to care for each patient’s physical and psychological safety, comfort, and dignity;
- keep patients’ medical and personal information confidential;
- provide for and facilitate the preoperative evaluation and informed decision making;
- convey possible IONM-P responsibility sharing;
- remain continuously available for direction and supervision of IONM-Ts perioperatively and fully participate in the most demanding aspects of IONM.

## Appendix

### General IONM-P methodology Guidelines

#### Preoperative

*An institution*-*specific IONM Policy and Procedure Manual is followed. A designee of the IONM monitoring team, under IONM*-*P supervision:*

1. Ensures that the credentials of the IONM-T(s) and IONM-P are up to date and the appropriate state, local, and hospital privileges are current.
2. Assigns one ongoing case at a time to each IONM-T.
3. Ensures that all equipment is currently up-to-date with appropriate software, and that hardware maintenance has been tested by the relevant hospital electrical safety department.
4. Ensures that all electrodes to be placed in the patient are sterile, and that the equipment is cleaned prior to each procedure.
5. Reviews the patient’s chart and records the following information:
  - The surgical/procedure planned.
  - The pre-procedural diagnosis and relevant neural co-morbidities, including previous procedures on or affecting the nervous system.
  - Significant medical diagnoses and relevant laboratory information, especially the presence of infectious agents that might contaminate the monitoring equipment.
  - The neurological findings on physical examination.
  - The findings on relevant studies (imaging, e.g.,) of the nervous system.
  - Latex or other allergies, if present
6. Discusses the case as needed with the surgeon or proceduralist to review the planned procedure and the relevant neural anatomy and physiology in order to determine the appropriate modalities to be monitored.
7. Reviews the preferred anesthetic regimens corresponding to the planned monitoring modalities with the anesthesiologist to promote optimized maintenance anesthesia conditions.
8. Discusses, as needed, the planned monitoring with the nursing staff in the procedure room to identify a suitable location for the monitoring equipment and IONM personnel.
9. Identifies IONM personnel to the patient to indicate their presence in the procedure. Any remaining questions related to neuromonitoring are answered as needed. Written consent is obtained if required by the hospital (absent any pre-op anesthesia).
10. The IONM-T(s) and the IONM-P will share the above information prior to the procedure (See, Sect. 6.3).

#### Intraoperative

*An institution*-*specific IONM Policy and Procedure Manual is followed. A designee of the IONM monitoring team, under IONM*-*P supervision:*

1. Ensures, under non-urgent and usual conditions, that IONM personnel arrive sufficiently before the procedure so that all equipment can be set-up in the surgery/procedural room before the patient comes into the room.
2. Ensures placement of electrodes (or other monitoring devices), using aseptic techniques and suitable skin preparation as defined by hospital policies.
3. Acquires baseline responses for all monitoring modalities as soon as possible or as appropriate.
4. Facilitates communication in real time between IONM personnel using HIPAA compliant techniques. Ongoing communication between the IONM personnel as it relates to patient care should be documented.
5. Informs the surgeon or proceduralist and the anesthesiologist of baseline IONM recordings. The IONM-T and IONM-P discuss alert criteria.
6. Acquires IONM responses based on the demands of the procedure. Discusses testing strategy with the surgeon or proceduralist to coordinate the IONM as needed.
7. Maintains a log of the monitoring:
  - Major findings of responses at various times.
  - Associated temperature, blood pressure and significant physiological data as available.
  - Comments about the stage of the surgery/procedure
  - Important communications with the operating room team and between the IONM-P and IONM-T.
8. Archives baseline responses, responses when an alert occurs, or significant milestones, and final responses.
9. Documents ongoing communication between IONM personnel. The IONM-P will be rapidly available to discuss recording interpretation/diagnosis/therapeutic recommendation with the surgeon or proceduralist and/or the anesthesiologist.

#### Postoperative

*An institution*-*specific IONM Policy and Procedure Manual is followed. A designee of the IONM monitoring team, under IONM*-*P supervision:*

1. Reviews reported clinical results of all monitored procedures.
2. Ensures IONM-P availability (personally or virtually) to consult with the surgeon or proceduralist and/or the anesthesiologist as indicated regarding the immediate assessment of or approach to any post-operative neural complications.
3. Determines if there were any quality assurance problems that should be more thoroughly reviewed, reported, or corrected.
4. Issues a patient chart report in accordance with hospital policy, including:
  - The patient identifying information, date of service, surgeon or proceduralist, and procedure performed, and names of IONM-T and IONM-P.
  - Monitoring modalities used and baseline data.
  - Comments about the baseline responses and comments regarding the monitoring that was done, including alerts and significant data points acquired during the procedure (e.g., pedicle screw thresholds).
  - A note regarding the final responses acquired.
5. Saves all data (including the final report) in a HIPAA compliant method. Records should be maintained for a period of time in accordance with applicable State law and/or recommendations of the applicable State medical board.

### IONM-P qualifications

An IONM-P holds an advanced academic (doctoral) degree. (MD, DO, PhD, AuD, for example). The IONM-P is most frequently credentialed by any one the following Boards: American Board of Neurophysiological Monitoring, American Board of Clinical Neurophysiology, American Board of Electroneurodiagnostic Medicine, American Board of Psychiatry and Neurology with added qualification in Clinical Neurophysiology. However, many Board-certified anesthesiologist, surgeon or proceduralist, and neurologist IONM-Ps, without specific certification by recognized IONM Boards, have contributed significantly to IONM literature/education/practice and will, in the near-term, continue to appropriately serve as qualified IONM-Ps.

In the future, specific certification by an IONM-related Board for all practicing IONM-Ps is expected. Currently, certification by the American Board of Neurophysiological Monitoring (ABNM) is the only nationally recognized certification for non-physician IONM-Ps.

The IONM-P must hold credentials to provide IONM supervision at all hospitals within their IONM practice. Before credentialing or re-credentialing, hospitals are strongly encouraged to demand evidence of an appropriate combination of board certification or re-certification, training, experience, and continuing education. For example, the hospital may require proof from the IONM-P candidate of specific didactic instruction, practical training, and evaluation in basic and applied neurophysiology (specific IONM modalities) during the initial credentialing process. The practical training could consist, for example, of a minimum of 50 cases supervised by an experienced IONM-P (tutor/mentor/training program director). The mentoring IONM-P must be either Board-certified (ABNM, ABCN, ABEM, or ABPN with added qualification in Clinical Neurophysiology) or acknowledged by the IONM community as an authority (evidenced by lecture presentations at regional or national IONM meetings/symposia, publications, or a Fellowship designation by the ASNM). Within the limits of each candidate’s scope of practice, the candidate IONM-P must demonstrate proficiency in areas of IONM detailed in these Guidelines: technological, interpretive, and interventional. Letters from surgeons or proceduralists attesting to these skills are highly recommended. The hospital may choose to limit the scope of practice to those kinds of cases for which the candidate IONM-P received specific training. Once in practice, the IONM-P is expected to actively supervise at least 250 cases every 3 years until a cumulative case experience of 1,000 cases is achieved. At this experience level, the case requirement may be decreased to permit active teaching and administration. Once in practice, at least 50 h of AMA Category I continuing education in clinical neurophysiology for the prior 3 years must be documented. The IONM-P’s certifying IONM-related Board may require more than this recommended continuing education minimum.

### Telemedicine considerations

Fully realized telemedicine implies a “patient care” duty that matches the obligation when physically present. During online IONM supervision, fully realized telemedicine (virtual presence) permits (as needed or appropriate):

1. Patient history taking, assisted examination/radiological image review, explanation of IONM procedures, and intraoperative waveform analysis;
2. IONM evaluation of patient positioning, lead placement, and images taken directly from the wound or through the operating microscope; and
3. Full two-way audiovisual communication between the on- or off-site IONM-P and the on-site caregivers: IONM-T, surgeon or proceduralist, and anesthesiologist.

### IONM protocols (IONM policy and procedure manual)

There are a number of sources that describe appropriate protocols for performing basic neurophysiological testing in the operating room. It is the responsibility of the IONM-P to understand and synthesize the data presented in these Guidelines as well as the peer reviewed literature in order to formulate an IONM Policy and Procedure Manual. Some helpful resources include:

American Clinical Neurophysiology Society (ACNS) Guidelines can be found at www.acns.org. Not every guideline has information specific to neurophysiological testing in the operating room but they do indicate good practices and are a valuable guide.

American Society for Neurophysiologic Monitoring (ASNM) Guidelines have been published for Somatosensory Evoked Potentials, Auditory Evoked Potentials, EMG/reflex studies, Motor Evoked Potentials, Intra-Operative EEG, and Transcranial Doppler. These are valuable guides to best practices. Additional Guidelines will be made available in the future. ASNM Guidelines can be found on the ASNM website at: www.asnm.org/Statements and at the ASET website: www.aset.org.
